# Prompt control of a *Serratia marcescens* outbreak in a neonatal intensive care unit informed by whole-genome sequencing and comprehensive infection control intervention package

**DOI:** 10.1017/ash.2022.234

**Published:** 2022-06-27

**Authors:** Annaleise R. Howard-Jones, Catherine Janto, Zoe Jennings, James Branley, Qinning Wang, Vitali Sintchenko, Harsha Samarasekara

**Affiliations:** 1 New South Wales Health Pathology-Nepean, Penrith, New South Wales, Australia; 2 Faculty of Medicine and Health, University of Sydney, Camperdown, New South Wales, Australia; 3 Department of Microbiology and Infectious Diseases, Nepean Hospital, Kingswood, New South Wales, Australia; 4 Centre for Infectious Diseases & Microbiology Laboratory Services, New South Wales Health Pathology–Institute of Clinical Pathology & Medical Research, Westmead, New South Wales, Australia

## Abstract

**Objective:**

This report describes a cluster of patients infected by *Serratia marcescens* in a metropolitan neonatal intensive care unit (NICU) and a package of infection control interventions that enabled rapid, effective termination of the outbreak.

**Design:**

Cross-sectional analytical study using whole-genome sequencing (WGS) for phylogenetic cluster analysis and identification of virulence and resistance genes.

**Setting:**

NICU in a metropolitan tertiary-care hospital in Sydney, Australia.

**Patients:**

All neonates admitted to the level 2 and level 3 neonatal unit.

**Interventions:**

Active inpatient and environmental screening for *Serratia marcescens* isolates with WGS analysis for identification of resistance genes as well as cluster relatedness between isolates. Planning and implementation of a targeted, multifaceted infection control intervention.

**Results:**

The cluster of 10 neonates colonized or infected with *Serratia marcescens* was identified in a metropolitan NICU. Two initial cases involved devastating intracranial infections with brain abscesses, highlighting the virulence of this organism. A targeted and comprehensive infection control intervention guided by WGS findings enabled termination of this outbreak within 15 days of onset. WGS examination demonstrated phylogenetic linkage across the cluster, and genomic unrelatedness of later strains identified in the neonatal unit and elsewhere.

**Conclusions:**

A comprehensive, multipronged, infection control package incorporating close stakeholder engagement, frequent microbiological patient screening, environmental screening, enhanced cleaning, optimization of hand hygiene and healthcare worker education was paramount to the prompt control of *Serratia marcescens* transmission in this neonatal outbreak. WGS was instrumental in establishing relatedness between isolates and identification of possible transmission pathways in an outbreak setting.

*Serratia marcescens* has emerged as a recognized cause of late-onset sepsis among premature and low-birth-weight neonates.^1^ Ability to thrive in a hospital environment, resistance to commonly used empirical antibiotics, and an array of virulence factors has enabled *S. marcescens* to emerge in nosocomial outbreak settings, particularly among immunocompromised patients in intensive care units and neonatal intensive care units (NICUs).^2^ In the neonatal population, risk factors for *S. marcescens* infection include immunological immaturity, low birth weight (<1,500 g), prolonged hospital stay, use of empiric antibiotics,^3^ and indwelling devices or instrumentation.^4^

*S. marcescens* can cause a broad spectrum of clinical syndromes, from asymptomatic colonization of the gastrointestinal tract or conjunctiva to urinary tract infections, pneumonia, or bloodstream infections.^5^ Clinically severe and fulminant central nervous system (CNS) complications, such as meningitis and cerebral abscesses have been well recognized.^6^ Invasive infections due to *S. marcescens* carry mortality rates of up to 45%.^3^

*S. marcescens* accounts for >110 published outbreaks worldwide,^7,8^ with 48% of these occurring in neonatal units.^8^ The source of the outbreaks often remains cryptic, with one study identifying a point source in only 40% of outbreaks.^1^ Contributing factors have been postulated to include contamination of products, equipment or sinks, inadequately disinfected surfaces, and poor hand hygiene among healthcare workers.^3,9–11^ Few of these outbreak investigations have utilized whole-genome sequencing (WGS) to describe cluster relatedness and possible transmission pathways,^2,5,12^ though the value of this approach appears to remain high in unravelling detailed resistance and transmission dynamics.

In this study we describe an outbreak investigation of *S. marcescens* in a metropolitan NICU in which WGS-based cluster identification was used to infer phylogenetic strain relatedness and to suggest termination of the outbreak. Furthermore, we present a successful package of infection control interventions implemented to terminate the outbreak and discuss contributors and useful insights for other neonatal units.

## Methods

### Study design

This cross-sectional study of *S. marcescens* isolates from clinical and environmental specimens obtained from a level 2 and 3 NICU was conducted in metropolitan Sydney, Australia, from April 19, 2018, to May 31, 2018.

### *Patient screening for* Serratia marcescens *colonization*

After identification of an initial 3 *S. marcescens* clinical isolates, active patient surveillance was initiated with twice weekly eye and rectal swabs (Copan Amies swab, Copan Diagnostics, Murrieta, CA) on all inpatients in the NICU. Screening was also conducted on all babies discharged from the NICU to the postnatal wards or home in the 48 hours prior to outbreak detection.

### *Environmental screening for* Serratia marcescens

Screening of environmental surfaces and equipment was conducted by surface swabbing (Copan Amies swab) at a single time point. For alcohol-based hand rubs, moisturizing solutions, and intravenous fluids, 5 mL of each solution was sampled. Breast milk was analyzed only as a pooled specimen (combined from all storage packs in the breast milk fridge at a single time point). Water sampling was conducted by filtration of a 300 mL sample from each tap or shower head through a 0.05 µm bacterial filter. Air sampling of the NICU was conducted using settle plates (Brilliance-UTI agar, Oxoid, Basingstoke, UK). Plates were left uncovered for 60 minutes then incubated for 18–24 hours at 37°C.

### Healthcare worker screening

Anonymous, voluntary staff screening was conducted using self-collected hand swabs and rectal or perianal swabs (Copan Amies swab). Swabs were cultured as described below.

### Culture-based methods

All swabs from patients (eye swabs, rectal swabs, clinical specimens), staff, and environmental surfaces were analyzed for the presence of *S. marcescens*. Swabs were inoculated within 4 hours of collection directly onto Brilliance-UTI agar (Oxoid) and incubated for 18-24 hours at 37 °C. Fluid specimens (5 mL) were inoculated into nutrient broth (brain heart infusion broth, 20 mL) and incubated for 16–18 hours at 37°C. A 10-µL sample of this broth was then used to inoculate Brilliance-UTI agar (Oxoid) and incubated at 37°C for 18–24 hours. Turquoise blue colonies were identified by MALDI-TOF analysis (Bruker Daltonics, Billerica, MA) and susceptibility testing performed by VITEK 2 (bioMérieux, Marcy-l’Étoile, France) using Clinical and Laboratory Standard Institute (CLSI) guidelines. Bacterial filters (0.05 µm) from water sampling processes were placed directly on horse blood agar and incubated at 37°C for 24–48 hours for bacterial growth. Any colonies grown were identified by MALDI-TOF analysis (Bruker Daltonics, Germany) as described above.

### WGS and data analysis

All isolates were subjected to WGS at the Centre for Infectious Diseases & Microbiology Laboratory Services (CIDMLS), Institute of Clinical Pathology & Medical Research (ICPMR), New South Wales Health Pathology, using the NextSeq 500 platform (Illumina) with libraries prepared using Nextera XT DNA Library Prep Kit (Illumina). The sequencing data quality was checked using an in-house procedure. SPAdes version 3.11.0 software was employed for de novo assembly and species identification was determined using Kraken version 0.10.5-beta software. Single nucleotide polymorphisms (SNPs) were identified using the Nullabor pipeline (https://github.com/tseemann/nullarbor). For SNP identification, reads were mapped to the *S. marcescens* reference genome of strain UMH12 (NCBI GenBank Accession CPO 18930.1). SNPs were defined as substitutions observed in at least 90% reads with a minimal coverage of 30 in depth. Core genome SNPs from each sequence were collected using Snippy version 3.0 software and the core SNP phylogeny was examined by generation of maximum likelihood tree using FastTree version 2.17 software. The antibiotic resistance and virulence gene profiles were inferred using ABRicate version 0.7 software with data from ResFinder and VFDB databases.

## Results

### Clinical setting

**Fig. 1.**
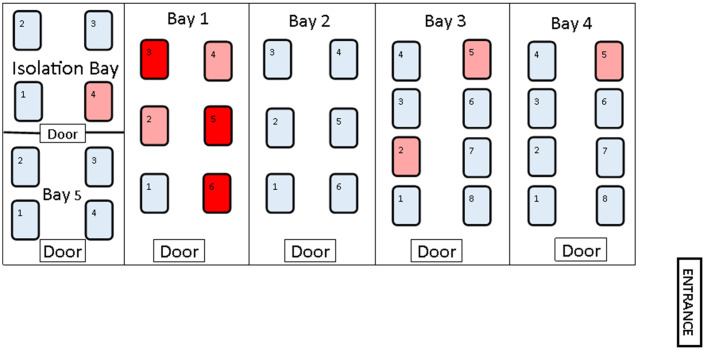
Schematic demonstrating layout of the 36 beds in the NICU, arranged in 4- to 8-bed bays. Cases with clinical *Serratia marcescens* infection at onset of the outbreak are indicated in red; cases with *S. marcescens* colonization at onset of the outbreak are indicated in pink; noncolonized babies are indicated in light blue.

The NICU included in the current study is a 36-bed facility accepting level 2 and 3 admissions from in-hospital deliveries and transfers from across the State. The unit comprises several 4-, 6- and 8-bed bays (Fig. 1) with 2 wet sinks within the unit.

### Outbreak detection

*S. marcescens* was isolated from 3 clinical specimens over 3 days, all from neonates cared for concurrently in a single bay of the NICU (Fig. 1, bay 1).

Case 1 was an ex–27-week female neonate who developed sepsis on day 9 of admission, with *S. marcescens* (susceptible to cefepime, meropenem, and gentamicin) isolated from blood cultures (Table 1, isolate 1). The neonate developed multiple brain abscesses on head ultrasound and died on day 11 from sepsis and multiorgan failure despite appropriate antibiotic therapy (IV cefepime from day 2 then meropenem from day 3).

**Table 1.** Characteristics of *Serratia marcescens* Clinical Isolates From the Neonatal Intensive Care Outbreak

Case	Isolate	WGS ID	Date of Culture	Sex	Gestational Age at Birth, weeks	Days from NICU admission to *Serratia marcescens* Isolation	Site of Isolation	Clinical Infection
1	1	18-0165-0002	19/04/2018	F	27	9	Blood culture	Bacteremia, brain abscess
2	2	18-0165-0001	20/04/2018	M	30	8	Eye swab	Brain abscess
2	3	18-0165-0011	04/05/2021	M	30	22	Brain abscess fluid	Brain abscess
3	4	18-0165-0007	22/04/2018	F	38	3	Rectal swab	Nil
4	5	18-0165-0003	22/04/2018	M	28	41	Rectal swab	Nil
5	6	18-0165-0004	22/04/2018	M	30	13	Rectal swab	Nil
6	7	18-0165-0005	22/04/2018	F	27	38	Rectal swab	Nil
7	8	18-0165-0006	22/04/2018	F	36	2	Rectal swab	Nil
8	9	18-0165-0008	22/04/2018	F	29	14	Eye swab	Nil
9	10	18-0165-0009	30/04/2018	F	25	43	Rectal swab	Nil
10	11	18-0165-0010	25/04/2018	M	36	2	Rectal swab	Nil

Note. WGS, whole-genome sequencing; ID, identifier; NICU, neonatal intensive care unit.

The following day, an ex–30-week neonate in the same bay (case 2) developed conjunctival discharge followed by seizures. Head ultrasound demonstrated a left parietal brain abscess which later ruptured into the lateral ventricle and required neurosurgical intervention. Although blood cultures were negative, *S. marcescens* was grown from both purulent eye discharge and brain abscess fluid (Table 1, isolates 2 and 3, respectively). The child survived but with significant neurological sequelae.

Case 3 was a full-term baby nursed in the same bay noted to be colonized with *S. marcescens* on rectal swab 3 days after the diagnosis of case 1. Although the child developed conjunctivitis and signs of sepsis with respiratory distress on day 3 of life, *S. marcescens* was not isolated from blood cultures nor eye swabs in this case. The child received IV flucloxacillin and gentamicin then meropenem for 7 days and recovered.

Based on review of these initial cases, an outbreak of *S. marcescens* was declared in the NICU, and the public health unit and hospital governance teams were informed.

### Infection prevention and control intervention planning

Declaration of the outbreak lead to formation of an outbreak management team with consultative advice from a public health specialist. Outbreak management team meetings were conducted at outbreak detection and at regular intervals (initially daily until no new cases identified and the infection prevention and control (IPC) package of interventions had been implemented) then with reduced frequency until end of the outbreak was declared.

### Implementation of infection prevention and control package

The acuity of the initial *S. marcescens* cases necessitated rapid escalation in the form of a carefully targeted package of IPC measures to promptly terminate the outbreak. The package comprised twice weekly active screening for *S. marcescens* colonization (as outlined below), active environmental screening throughout the NICU (as detailed below), voluntary staff screening, education of healthcare workers and parents, and optimization of hand hygiene.

All neonates within the unit were managed under contact precautions until a negative rectal and eye swab result was confirmed. If *S. marcescens* colonization was detected, contact precautions were continued throughout the admission with cohorting of colonized babies in dedicated patient bays. With declaration of the outbreak, a satellite nursery was prepared following deep cleaning in a physically separate location for new admissions and for existing NICU patients ≥35 weeks gestation. Additional medical and nursing staff were deployed to care for neonates in this unit.

An educational fact sheet was prepared including the nature of the organism, mode of spread, types of infections caused, antibiotic options and infection control precautions to prevent the spread of the outbreak. This information was communicated to healthcare workers during dedicated staff meetings and was provided in written form to parents and, when required, via individual meetings with concerned caregivers.

All sinks, water supply, drains, and plumbing were examined by the hospital maintenance engineer for cracks and crevices which might harbor bacteria, and no major defects were identified. Sinks were deep cleaned twice daily using 10% hypochlorite solution for the duration of the outbreak.

The status of hand hygiene compliance at the time of the outbreak was reviewed closely by the IPC team with a view to identifying opportunities for improvement. Over the preceding 12 months, overall compliance rates with hand hygiene had been 90.6% (Supplementary Fig. S1). Hand hygiene education sessions for healthcare workers were increased to twice weekly for medical and nursing staff, and hand hygiene audits (including spot audits) were performed more frequently. Hand hygiene compliance increased to 95.3% in the 12 months following implementation of these interventions; this rate of compliance was maintained for 2.5 years after the intervention (to December 2021, data not shown).

### Active patient screening for colonization and review of historical cultures

One of the neonates present in the NICU during this outbreak (case 4) was known to be colonized with *S. marcescens* from an eye swab 30 days prior. Unfortunately, the initial isolate was not available for comparative sequencing, but *S. marcescens* was again isolated from a rectal swab of this patient during the outbreak period (Table 1, isolate 5). Furthermore, 26 patients were screened in the initial point-prevalence survey when the outbreak was detected, and 4 additional cases of *S. marcescens* colonization were detected among neonates still cared for within the NICU. No cases were identified in babies on the postnatal wards or those discharged home. Twice weekly surveillance of all NICU inpatients revealed an additional 2 cases in the subsequent 11 days, bringing the total number of cases to 10, with no cases identified in the 4 weeks thereafter (Table 1 and Supplementary Fig. S2).

### Results of environmental screening

In total, 284 environmental samples were obtained prior to implementation of enhanced cleaning procedures. These samples were taken from all clinical areas of the NICU: sinks and water sources (n = 33), frequently touched surfaces (n = 61), equipment and items frequently in contact with neonates (n = 18), hygiene-related products (alcohol hand rubs, moisturizing solutions, liquid soaps and single use nonsterile gloves) (n = 91), stores of unused intravenous fluids (n=8), items related to breastmilk management (breast milk pumps, milk storage bags, breast milk warmers, high-touch surfaces of the breast milk fridges, pooled breast milk sample) (n = 29), and air sampling in the NICU using settle plates (n=8), as well as frequently touched surfaces and equipment (n = 23) and resuscitation equipment (n = 13) in the postnatal ward and theaters. *S. marcescens* was not isolated from any of these 284 environmental samples. Breastmilk-related samples grew either no organisms or mixed skin flora, and intravenous fluids were sterile.

### Voluntary healthcare worker screening

Because all other environment samples failed to yield a point source, staff screening was implemented as a last resort option and on a voluntary basis. All samples were anonymized for laboratory processing to maintain confidentiality. Only 10% of the NICU staff participated in the screening process, yielding 9 hand swab cultures and 8 perianal or rectal swabs. None of the staff swab cultures yielded *S. marcescens.*

### Virulence and relatedness assessment by whole-genome sequencing

In total, 11 *S. marcescens* isolates from 10 NICU patients during the outbreak period were subjected to WGS and comparative genomics analysis. In addition, 3 *S. marcescens* isolates identified in the NICU over 1 month later and 4 further clinical *S. marcescens* sterile site isolates from other wards in our institution (3 isolates) and an external hospital (1 isolate) were submitted for comparison.

**Fig. 2.**
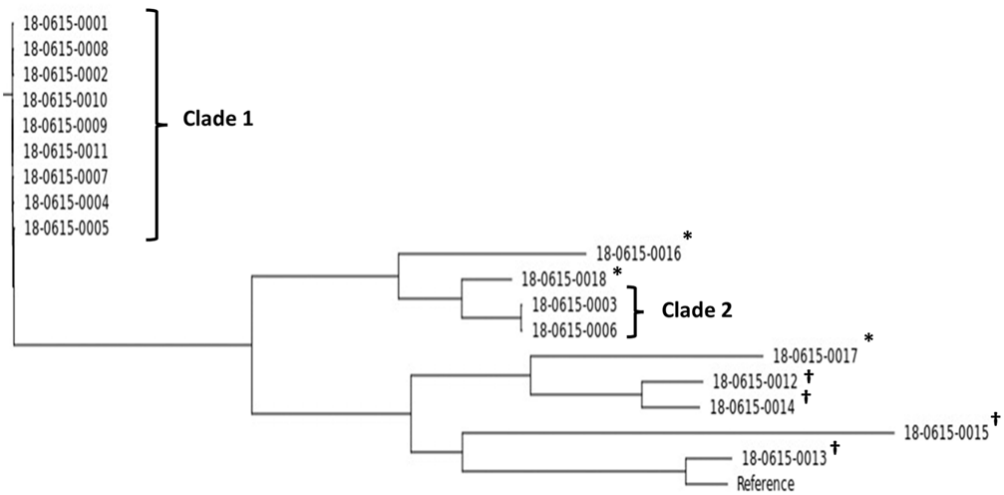
Phylogenetic analysis of single nucleotide polymorphisms (SNPs) in 11 isolates of *Serratia marcescens* from the NICU outbreak, with 4 comparator strains from other locations (highlighted with †). The 3 postoutbreak strains are highlighted with asterisks, each belonging to a distinct clade.

All sequenced isolates were identified as *S. marcescens* by WGS. The SNP analysis performed on the WGS data demonstrated that the 11 clinical isolates from the NICU cluster belonged to 2 distinct clades, separated by >110,000 SNPs. Clade 1 consisted of 9 *S. marcescens* strains with a range of 3–48 SNPs within the genomes, including those from the 2 patients with confirmed invasive disease (cases 1 and 2; isolates 1, 2, and 3) and the putative source case (case 4, isolate 5). The remaining 2 outbreak strains belonged to a second clade (clade 2), and were separated from each other by 28 SNPs (Supplementary Table S3). The 4 unrelated isolates from other wards in our institution and an external hospital were genomically distinct from these 2 clades (Fig. 2) by a minimum of ∼30,000 SNPs. Moreover, the 3 NICU isolates that emerged following termination of the outbreak (days 36–39) were distinct to the outbreak strains and to each other (Fig. 2, highlighted with an asterisk).

In total, 19 putative virulence factors were predicted by the virulence factor database (VFDB) in the predominant majority of genomes of the *S. marcesens* isolates, which mainly accounted for flagella formation, chemotaxis, motor protein, and outer membrane protein. The virulence genes inferred from WGS data had no discernible difference to separate the 11 NICU isolates from the other genomes sequenced (Supplementary Table S1).

The antimicrobial resistance profiles inferred from WGS data grouped the 11 genomes from the NICU into 2 AMR clusters with the same sequence composition as that identified by SNP analysis (Supplementary Table S2). The genomes of isolates from other origins presented various AMR profiles that were distinct from these 2 clades.

### Declaration of the end of the outbreak

The outbreak encompassed 10 patients, 2 with profound *S. marcescens*–related clinical disease (brain abscess with and without bacteremia) and 8 with colonization (1 of these with clinical concern for sepsis but no sterile site isolate). No positive environmental or staff screening samples were identified. Twice weekly patient surveillance for *S. marcescens* colonization was continued for a total of 8 weeks, followed by routine weekly surveillance indefinitely. The outbreak was declared terminated after a period of 21 days with no new detection of *S. marcescens* colonization or clinical infection within the unit despite regular surveillance.

## Discussion

*Serriatia marcescens* is a common cause of nosocomial outbreaks, particularly in neonatal units.^1^ The outbreak reported herein, with 2 devastating cases of CNS infection, highlights the importance of minimizing nosocomial spread of virulent organisms within a vulnerable NICU population.

*S. marcescens* is well known to spread by contaminated hands, leading to outbreaks in NICUs.^3,5,10^ At our institution, the NICU has been one of the most compliant units when audited against the WHO Five Moments of Hand Hygiene.^13^ Optimizing hand hygiene was an important part of the multipronged intervention to terminate this outbreak, in line with established data.^3,5,10^ Resultant compliance rates of 95% were maintained for over 2 years after the intervention, suggesting long-term efficacy of hand hygiene measures with appropriate healthcare worker engagement.

Central to the effective termination of this outbreak was key stakeholder engagement, incorporating close communication with the local public health department, hospital management, healthcare staff, and parents. Due to the clinical severity of the initial cases, there was significant concern about this organism among parents and healthcare workers. Effective communication and targeted education materials ensured engagement by all parties in controlling this outbreak in a prompt and safe manner. The comprehensive IPC package intervention implemented in this report enabled rapid termination of this outbreak involving 10 neonatal cases within 15 days, as compared to an average of 27 cases for previously published *S. marcescens* outbreaks.^7^ The bundled measures included early and frequent microbiological screening to identify colonized or infected neonates, contact precautions (with cohorting as required), investigation for potential environmental sources, enhanced cleaning of surfaces, optimization of hand hygiene, healthcare worker education and continuous ongoing microbiological surveillance. The prompt control of transmission using this IPC package carries many salient lessons for IPC in NICU departments worldwide to prevent or rapidly contain future outbreaks of a similar nature.

The use of WGS was invaluable in this outbreak evaluation in informing cluster relatedness including possible transmission pathways and in identifying characteristic antimicrobial resistance profiles and virulence genes of concern. Two distinct SNP clusters were identified in the 11 genomes from the NICU, which were different from the other genomes acquired from other origins. The presence of 2 separate *S. marcescens* clades in this investigation was of concern, highlighting the critical need for introduction of a targeted IPC package to ameliorate risk of future introductions into this ward. The genomic AMR profiles inferred from the WGS data further supported the SNP-based clustering analysis and were consistent with the expected epidemiology of this cluster. Virulence gene profile analysis was not able to provide distinct gene signatures for the strains involved in the outbreak, which may indicate evolutionary conservation of the virulence genes in this species. In well-resourced settings, future infection control efforts should aim to incorporate WGS strategies to enable such powerful analyses and to guide IPC strategies at the ward and population levels.

In conclusion, our comprehensive, multipronged, infection control package incorporating close stakeholder engagement, frequent microbiological patient screening, environmental screening, enhanced cleaning, optimization of hand hygiene and healthcare worker education, was paramount to the prompt control of *S. marcescens* transmission in this neonatal outbreak. WGS was instrumental in establishing relatedness between isolates and identification of possible transmission pathways in an outbreak setting.

## Supplementary material

For supplementary material accompanying this paper visit https://doi.org/10.1017/ash.2022.234. click here to view supplementary material

## References

[1] Gastmeier P. *Serratia marcescens*: an outbreak experience. Front Microbiol 2014;5:81. 24639671 10.3389/fmicb.2014.00081 PMC3944479

[2] Martineau C , Li X , Lalancette C , Perreault T , et al. *Serratia marcescens* outbreak in a neonatal intensive care unit: new insights from next-generation sequencing applications. J Clin Microbiol 2018;56:e00235–18. 29899005 10.1128/JCM.00235-18 PMC6113457

[3] Cristina ML , Sartini M , Spagnolo AM. *Serratia marcescens* infections in neonatal intensive care units (NICUs). Int J Environ Res Public Health 2019;16:2648. 30791509 10.3390/ijerph16040610 PMC6406414

[4] Samuelsson A , Isaksson B , Hanberger H , Olhager E. Late-onset neonatal sepsis, risk factors and interventions: an analysis of recurrent outbreaks of *Serratia marcescens*, 2006–2011. J Hosp Infect 2014;86:57–63. 24332914 10.1016/j.jhin.2013.09.017

[5] Zingg W , Soulake I , Baud D , et al. Management and investigation of a *Serratia marcescens* outbreak in a neonatal unit in Switzerland—the role of hand hygiene and whole-genome sequencing. Antimicrob Resist Infect Control 2017;6:125. 29238572 10.1186/s13756-017-0285-x PMC5725813

[6] Hirooka TM , Fontes RB , Diniz EM , Pinto FC , Matushita H. Cerebral abscess caused by *Serratia marcescens* in a premature neonate. Arquivos de Neuro-psiquiatria 2007;65:1018–1021. 18094868 10.1590/s0004-282x2007000600021

[7] Gastmeier P , Loui A , Stamm-Balderjahn S , et al. Outbreaks in neonatal intensive care units—they are not like others. Am J Infect Control 2007;35:172–176. 17433940 10.1016/j.ajic.2006.07.007

[8] Worldwide database for nosocomial outbreaks. Outbreak Database website. https://www.outbreak-database.com/About.aspx. Published 2021. Accessed February 7, 2022.

[9] Fleisch F , Zimmermann-Baer U , Zbinden R , et al. Three consecutive outbreaks of *Serratia marcescens* in a neonatal intensive care unit. Clin Infect Dis 2002;34:767–773. 11830800 10.1086/339046

[10] Montagnani C , Cocchi P , Lega L , et al. *Serratia marcescens* outbreak in a neonatal intensive care unit: crucial role of implementing hand hygiene among external consultants. BMC Infect Dis 2015;15:11. 25582674 10.1186/s12879-014-0734-6 PMC4301457

[11] Rabier V , Bataillon S , Jolivet-Gougeon A , Chapplain JM , Beuchée A , Bétrémieux P. Handwashing soap as a source of neonatal *Serratia marcescens* outbreak. Acta Paediatr (Oslo, Norway). 2008;97:1381–1385. 10.1111/j.1651-2227.2008.00953.x 18782359

[12] Rohit A , Suresh Kumar D , Dhinakaran I , et al. Whole-genome–based analysis reveals multiclone *Serratia marcescens* outbreaks in a non-neonatal intensive care unit setting in a tertiary-care hospital in India. J Med Microbiol 2019;68:616–621. 30839251 10.1099/jmm.0.000947

[13] Sax H , Allegranzi B , Uçkay I , Larson E , Boyce J , Pittet D. “My five moments for hand hygiene”: a user-centred design approach to understand, train, monitor, and report hand hygiene. J Hosp Infect 2007;67:9–21. 17719685 10.1016/j.jhin.2007.06.004

