# Supplementary material for: Prompt control of a Serratia marcescens outbreak in a neonatal intensive care unit informed by whole-genome sequencing and comprehensive infection control intervention package
Source: Antimicrob Steward Healthc Epidemiol. 2022 Jun 27;2(1):e104. doi: 10.1017/ash.2022.234 (PMC9726519; doi:10.1017/ash.2022.234)
Supplement: Supplementary file 1 [file S2732494X22002340sup001.zip › S2732494X22002340sup001.docx]

**Supplementary Figure S1.** Compliance with hand hygiene amongst neonatal intensive care unit (NICU) health care workers in the 12 months prior to (2017-2018) and 12 months following the NICU *Serratia marcescens* outbreak (2018-2019). The yearly average compliance is indicated in the right-hand bars.
