# Supplementary material for: Prompt control of a Serratia marcescens outbreak in a neonatal intensive care unit informed by whole-genome sequencing and comprehensive infection control intervention package
Source: Antimicrob Steward Healthc Epidemiol. 2022 Jun 27;2(1):e104. doi: 10.1017/ash.2022.234 (PMC9726519; doi:10.1017/ash.2022.234)
Supplement: Supplementary file 1 [file S2732494X22002340sup001.zip › S2732494X22002340sup002.docx]

**Supplementary Table S3.** Differences in single nucleotide polymorphisms between isolates analysed as part of this cluster. Isolates from the neonatal intensive care cluster are highlighted in green.

|  | **18-0615-0001** | **18-0615-0002** | **18-0615-0003** | **18-0615-0004** | **18-0615-0005** | **18-0615-0006** | **18-0615-0007** | **18-0615-0008** | **18-0615-0009** | **18-0615-0010** | **18-0615-0011** | **18-0615-0012** | **18-0615-0013** | **18-0615-0014** | **18-0615-0015** | **18-0615-0016** | **18-0615-0017** | **18-0615-0018** | **Reference** |
| --- | --- | --- | --- | --- | --- | --- | --- | --- | --- | --- | --- | --- | --- | --- | --- | --- | --- | --- | --- |
| **18-0615-0001** | 0 | 45 | 119102 | 42 | 45 | 119107 | 33 | 33 | 34 | 33 | 40 | 148962 | 152731 | 147742 | 176954 | 130388 | 153242 | 117338 | 152519 |
| **18-0615-0002** | 45 | 0 | 119113 | 29 | 47 | 119118 | 18 | 20 | 19 | 18 | 27 | 148959 | 152747 | 147736 | 176956 | 130396 | 153238 | 117346 | 152534 |
| **18-0615-0003** | 119102 | 119113 | 0 | 119100 | 119104 | 28 | 119106 | 119107 | 119108 | 119105 | 119108 | 157521 | 153855 | 156170 | 180103 | 84281 | 161528 | 33778 | 153809 |
| **18-0615-0004** | 42 | 29 | 119100 | 0 | 48 | 119107 | 17 | 17 | 18 | 17 | 24 | 148957 | 152749 | 147737 | 176953 | 130389 | 153238 | 117334 | 152535 |
| **18-0615-0005** | 45 | 47 | 119104 | 48 | 0 | 119111 | 41 | 39 | 40 | 39 | 48 | 148958 | 152729 | 147735 | 176956 | 130392 | 153234 | 117339 | 152514 |
| **18-0615-0006** | 119107 | 119118 | 28 | 119107 | 119111 | 0 | 119111 | 119112 | 119113 | 119110 | 119113 | 157522 | 153855 | 156173 | 180101 | 84279 | 161535 | 33775 | 153813 |
| **18-0615-0007** | 33 | 18 | 119106 | 17 | 41 | 119111 | 0 | 8 | 7 | 6 | 15 | 148964 | 152758 | 147744 | 176961 | 130390 | 153244 | 117338 | 152546 |
| **18-0615-0008** | 33 | 20 | 119107 | 17 | 39 | 119112 | 8 | 0 | 7 | 6 | 15 | 148968 | 152762 | 147748 | 176965 | 130394 | 153248 | 117339 | 152550 |
| **18-0615-0009** | 34 | 19 | 119108 | 18 | 40 | 119113 | 7 | 7 | 0 | 3 | 14 | 148969 | 152760 | 147749 | 176963 | 130393 | 153245 | 117341 | 152547 |
| **18-0615-0010** | 33 | 18 | 119105 | 17 | 39 | 119110 | 6 | 6 | 3 | 0 | 13 | 148966 | 152761 | 147746 | 176964 | 130390 | 153246 | 117338 | 152548 |
| **18-0615-0011** | 40 | 27 | 119108 | 24 | 48 | 119113 | 15 | 15 | 14 | 13 | 0 | 148973 | 152758 | 147752 | 176964 | 130390 | 153251 | 117337 | 152545 |
| **18-0615-0012** | 148962 | 148959 | 157521 | 148957 | 148958 | 157522 | 148964 | 148968 | 148969 | 148966 | 148973 | 0 | 139961 | 36955 | 155438 | 167089 | 103043 | 157418 | 139557 |
| **18-0615-0013** | 152731 | 152747 | 153855 | 152749 | 152729 | 153855 | 152758 | 152762 | 152760 | 152761 | 152758 | 139961 | 0 | 136860 | 153353 | 166284 | 155722 | 155079 | 28773 |
| **18-0615-0014** | 147742 | 147736 | 156170 | 147737 | 147735 | 156173 | 147744 | 147748 | 147749 | 147746 | 147752 | 36955 | 136860 | 0 | 156075 | 165929 | 103362 | 156120 | 136318 |
| **18-0615-0015** | 176954 | 176956 | 180103 | 176953 | 176956 | 180101 | 176961 | 176965 | 176963 | 176964 | 176964 | 155438 | 153353 | 156075 | 0 | 188669 | 157718 | 180334 | 153017 |
| **18-0615-0016** | 130388 | 130396 | 84281 | 130389 | 130392 | 84279 | 130390 | 130394 | 130393 | 130390 | 130390 | 167089 | 166284 | 165929 | 188669 | 0 | 170516 | 81275 | 166219 |
| **18-0615-0017** | 153242 | 153238 | 161528 | 153238 | 153234 | 161535 | 153244 | 153248 | 153245 | 153246 | 153251 | 103043 | 155722 | 103362 | 157718 | 170516 | 0 | 161175 | 155443 |
| **18-0615-0018** | 117338 | 117346 | 33778 | 117334 | 117339 | 33775 | 117338 | 117339 | 117341 | 117338 | 117337 | 157418 | 155079 | 156120 | 180334 | 81275 | 161175 | 0 | 155037 |
| **Reference** | 152519 | 152534 | 153809 | 152535 | 152514 | 153813 | 152546 | 152550 | 152547 | 152548 | 152545 | 139557 | 28773 | 136318 | 153017 | 166219 | 155443 | 155037 | 0 |
