# Supplementary material for: Prompt control of a Serratia marcescens outbreak in a neonatal intensive care unit informed by whole-genome sequencing and comprehensive infection control intervention package
Source: Antimicrob Steward Healthc Epidemiol. 2022 Jun 27;2(1):e104. doi: 10.1017/ash.2022.234 (PMC9726519; doi:10.1017/ash.2022.234)
Supplement: Supplementary file 1 [file S2732494X22002340sup001.zip › S2732494X22002340sup003.docx]

**Supplementary Table S2.** Whole genome sequencing-based analysis of resistance genes present in *Serratia marcescens* isolates from the NICU outbreak. The isolates from Clade 1 are highlighted in light green, Clade 2 isolates in dark green and all other isolates are represented in white.

| **Isolate** | **Number of Genes Found** | **aac(6')** | **aac(6')-Ial** | **aac(6')-Ic** | **blaSRT** | **blaSRT-2** | **blaSST-1** | **catB1** | **oqxB10** | **oqxB30** | **oqxB5** | **oqxB9** | **qnrB27** | **qnrE1** | **tet(41)** |
| --- | --- | --- | --- | --- | --- | --- | --- | --- | --- | --- | --- | --- | --- | --- | --- |
| 18-0615-0001 | 5 | - | - | + | - | + | - | - | ± | - | - | + | - | + | - |
| 18-0615-0002 | 5 | - | - | + | - | + | - | - | ± | - | - | + | - | + | - |
| 18-0615-0003 | 7 | + | - | - | + | - | - | ± | ± | - | - | + | + | - | + |
| 18-0615-0004 | 5 | - | - | + | - | + | - | - | ± | - | - | + | - | + | - |
| 18-0615-0005 | 5 | - | - | + | - | + | - | - | ± | - | - | + | - | + | - |
| 18-0615-0006 | 7 | + | - | - | + | - | - | ± | ± | - | - | + | + | - | + |
| 18-0615-0007 | 5 | - | - | + | - | + | - | - | ± | - | - | + | - | + | - |
| 18-0615-0008 | 5 | - | - | + | - | + | - | - | ± | - | - | + | - | + | - |
| 18-0615-0009 | 5 | - | - | + | - | + | - | - | ± | - | - | + | - | + | - |
| 18-0615-0010 | 5 | - | - | + | - | + | - | - | ± | - | - | + | - | + | - |
| 18-0615-0011 | 5 | - | - | + | - | + | - | - | ± | - | - | + | - | + | - |
| 18-0615-0012 | 7 | - | + | - | - | - | + | ± | ± | - | + | - | - | + | + |
| 18-0615-0013 | 6 | + | - | - | + | - | - | - | ± | + | - | - | - | + | + |
| 18-0615-0014 | 6 | + | - | - | - | - | + | - | ± | - | + | - | - | + | + |
| 18-0615-0015 | 5 | + | - | - | - | - | + | - | - | - | + | - | - | + | + |
| 18-0615-0016 | 7 | + | - | - | + | - | - | ± | ± | - | - | + | - | + | + |
| 18-0615-0017 | 5 | + | - | - | - | - | + | - | - | - | - | + | + | - | + |
| 18-0615-0018 | 7 | + | - | - | + | - | - | ± | ± | - | - | + | ± | - | + |
