# Supplementary material for: Prompt control of a Serratia marcescens outbreak in a neonatal intensive care unit informed by whole-genome sequencing and comprehensive infection control intervention package
Source: Antimicrob Steward Healthc Epidemiol. 2022 Jun 27;2(1):e104. doi: 10.1017/ash.2022.234 (PMC9726519; doi:10.1017/ash.2022.234)
Supplement: Supplementary file 1 [file S2732494X22002340sup001.zip › S2732494X22002340sup004.docx]

**Supplementary Table S1.** Whole genome sequencing-based analysis of virulence genes present in *Serratia marcescens* isolates from the NICU outbreak. The isolates from Clade 1 are highlighted in light green, Clade 2 isolates in dark green and all other isolates are represented in white. No characteristic signatures in the virulence gene profiles were identified that could reliably differentiate the Clades.

| **Isolate** | **Number of Genes Found** | **aprA** | **cheA** | **cheB** | **cheD** | **cheR** | **cheW** | **cheY** | **clpV1** | **fimE** | **flgB** | **flgC** | **flgD** | **flgG** | **flgH** | **flgI** | **flhA** | **flhC** | **flhD** | **fliA** | **fliC** | **fliG** |
| --- | --- | --- | --- | --- | --- | --- | --- | --- | --- | --- | --- | --- | --- | --- | --- | --- | --- | --- | --- | --- | --- | --- |
| 18-0615-0001 | 33 | ± | ± | + | ± | + | + | + | ± | - | + | + | ± | + | ± | + | + | + | + | ± | ± | + |
| 18-0615-0002 | 34 | ± | ± | + | ± | + | + | + | ± | ± | + | + | ± | + | ± | + | + | + | + | + | ± | + |
| 18-0615-0003 | 35 | ± | ± | + | ± | + | + | + | ± | ± | + | + | ± | + | ± | + | + | + | + | + | ± | + |
| 18-0615-0004 | 34 | ± | ± | + | ± | + | + | + | ± | ± | + | + | ± | + | ± | + | + | + | + | ± | ± | + |
| 18-0615-0005 | 34 | ± | ± | + | ± | + | + | + | ± | ± | + | + | ± | + | ± | + | + | + | + | ± | ± | + |
| 18-0615-0006 | 35 | ± | ± | + | ± | + | + | + | ± | ± | + | + | ± | + | ± | + | + | + | + | + | ± | + |
| 18-0615-0007 | 33 | ± | ± | + | ± | + | + | + | ± | - | + | + | ± | + | ± | + | + | + | + | ± | ± | + |
| 18-0615-0008 | 34 | ± | ± | + | ± | + | + | + | ± | ± | + | + | ± | + | ± | + | + | + | + | + | ± | + |
| 18-0615-0009 | 34 | ± | ± | + | ± | + | + | + | ± | ± | + | + | ± | ± | ± | + | + | + | + | + | ± | + |
| 18-0615-0010 | 34 | ± | ± | + | ± | + | + | + | ± | ± | + | + | ± | + | ± | + | + | + | + | + | ± | + |
| 18-0615-0011 | 34 | ± | ± | + | ± | + | + | + | ± | ± | + | + | ± | + | ± | + | + | + | + | ± | ± | + |
| 18-0615-0012 | 28 | ± | ± | + | - | + | + | + | ± | - | + | + | - | + | ± | + | + | + | + | + | ± | + |
| 18-0615-0013 | 28 | ± | ± | - | - | + | + | + | ± | - | + | + | - | + | ± | + | + | + | + | + | ± | + |
| 18-0615-0014 | 30 | ± | - | + | ± | + | + | + | ± | - | + | + | - | + | ± | + | + | + | + | + | ± | + |
| 18-0615-0015 | 31 | - | ± | + | - | + | + | + | ± | ± | + | + | - | + | ± | + | + | + | + | + | ± | + |
| 18-0615-0016 | 34 | ± | ± | + | ± | + | + | + | ± | - | + | + | ± | + | ± | + | + | + | + | + | ± | + |
| 18-0615-0017 | 31 | ± | - | + | ± | + | + | + | - | ± | + | + | ± | + | + | + | + | + | + | + | ± | + |
| 18-0615-0018 | 36 | ± | ± | + | ± | + | + | + | ± | ± | + | + | ± | + | ± | + | + | + | + | + | ± | + |

| **Isolate (data continued)** | **fliH** | **fliI** | **fliL** | **fliM** | **fliN** | **fliP** | **fliQ** | **fliZ** | **gspG** | **hsiB1/vipA** | **hsiC1/vipB** | **iucD** | **mgtB** | **mgtC** | **motA** | **ompA** | **plcH** | **tssH-5/clpV** | **vgrG1b** | **yagZ/ecpA** |
| --- | --- | --- | --- | --- | --- | --- | --- | --- | --- | --- | --- | --- | --- | --- | --- | --- | --- | --- | --- | --- |
| 18-0615-0001 | - | + | - | + | + | + | + | + | ± | ± | ± | - | ± | - | + | + | - | - | ± | - |
| 18-0615-0002 | - | + | - | + | + | + | + | + | ± | ± | ± | - | ± | - | + | + | - | - | ± | - |
| 18-0615-0003 | ± | + | - | + | + | + | + | + | ± | - | ± | - | - | ± | + | + | ± | - | - | ± |
| 18-0615-0004 | - | + | - | + | + | + | + | + | ± | ± | ± | - | ± | - | + | + | - | - | ± | - |
| 18-0615-0005 | - | + | - | + | + | + | + | + | ± | ± | ± | - | ± | - | + | + | - | - | ± | - |
| 18-0615-0006 | ± | + | - | + | + | + | + | + | ± | - | ± | - | - | ± | + | + | ± | - | - | ± |
| 18-0615-0007 | - | + | - | + | + | + | + | + | ± | ± | ± | - | ± | - | + | + | - | - | ± | - |
| 18-0615-0008 | - | + | - | + | + | + | + | + | ± | ± | ± | - | ± | - | + | + | - | - | ± | - |
| 18-0615-0009 | - | + | - | + | + | + | + | + | ± | ± | ± | - | ± | - | + | + | - | - | ± | - |
| 18-0615-0010 | - | + | - | + | + | + | + | + | ± | ± | ± | - | ± | - | + | + | - | - | ± | - |
| 18-0615-0011 | - | + | - | + | + | + | + | + | ± | ± | ± | - | ± | - | + | + | - | - | ± | - |
| 18-0615-0012 | - | + | - | + | + | + | + | + | - | ± | ± | - | - | - | + | + | - | - | - | - |
| 18-0615-0013 | - | + | ± | + | + | + | + | + | - | - | ± | - | - | - | + | + | - | - | ± | - |
| 18-0615-0014 | - | + | - | + | + | + | + | + | - | ± | ± | - | ± | - | + | + | ± | - | - | - |
| 18-0615-0015 | - | + | - | + | + | + | + | + | ± | - | ± | - | - | - | + | + | ± | ± | ± | - |
| 18-0615-0016 | - | + | - | + | + | + | + | + | - | ± | ± | - | ± | - | + | + | ± | ± | - | ± |
| 18-0615-0017 | - | + | - | + | + | + | + | + | - | - | ± | - | ± | - | + | + | ± | - | - | ± |
| 18-0615-0018 | - | + | ± | + | + | + | + | + | ± | - | ± | ± | ± | - | + | + | ± | - | - | ± |
