# Supplementary material for: Prompt control of a Serratia marcescens outbreak in a neonatal intensive care unit informed by whole-genome sequencing and comprehensive infection control intervention package
Source: Antimicrob Steward Healthc Epidemiol. 2022 Jun 27;2(1):e104. doi: 10.1017/ash.2022.234 (PMC9726519; doi:10.1017/ash.2022.234)
Supplement: Supplementary file 1 [file S2732494X22002340sup001.zip › S2732494X22002340sup005.docx]

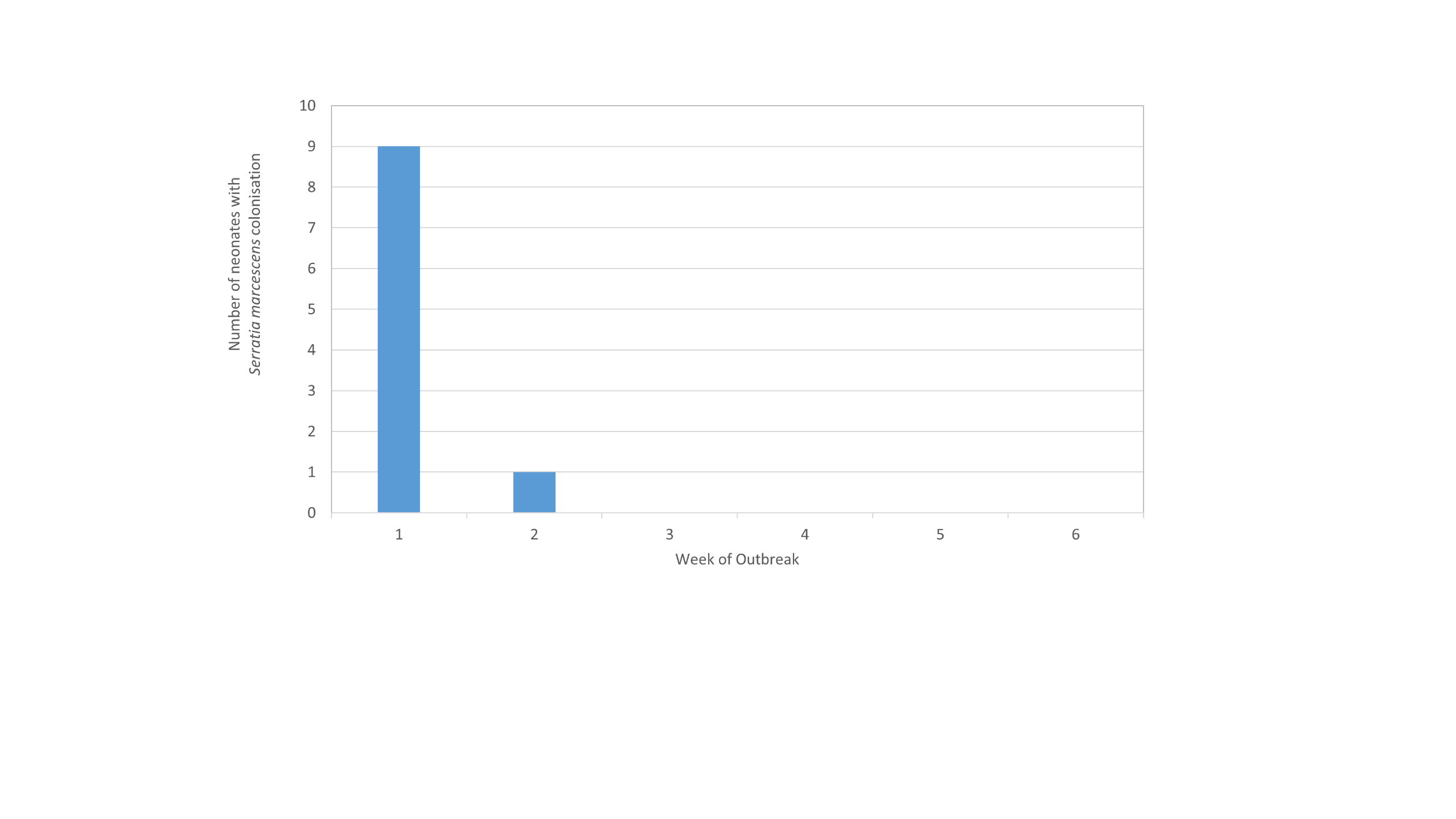


**Supplementary Figure S2.** Number of neonates with Serratia marcescens colonization by week of sampling.
